# The Classification, Correct Dietary, and Treatment of Diseases of the Skin, as Practised at the Belfast Hospital for the Skin

**Published:** 1889-09

**Authors:** 


*The Classification, Correct Dietary, and Treatment of Diseases of the Skin, as practised at the Belfast Hospital for the Skin.* By HENRY S. PURDON, M.D. London: BAILLIÈRÈ (*sic*), TINDALL AND COX. 1889.

For some time we were in doubt whether this work should be considered a serious book, or whether it could serve any useful purpose. Of the 32 pages of which it consists, 21 are arranged in double columns—one of which gives the cause and description of the disease, and the other its treatment; but about half the whole space is bare.

It must be said, however, that the suggestions for treatment, which offer a pleasing variety of applications, are on the whole sensible and devoid of "fads," if we except a manifest leaning towards vegetarianism. The writer might have given us a little more detail about the preparation of his "Pomades," and the specification of particular firms should have been avoided. Excellent "ærated" (*sic*) waters can be got without going to "the eminent firm of" Blank and Dash, and desirable preparations of cocoa and of white soup without necessarily patronising the establishments mentioned.

The book is closed with a drawing of the unpretentious-looking building in which the writer is gaining his experience. This, in days when we are anxious to know everything about the personal environment of a writer, has its interest; but it is difficult to see how it is to help the general reader striving to cure skin-diseases. Altogether, this is a style of book we do not like.

